# The complete chloroplast genome of *Saussurea medusa* maxim. (Asteraceae), an alpine Tibetan herb

**DOI:** 10.1080/23802359.2021.1984328

**Published:** 2021-10-07

**Authors:** Yongsheng Wei, Zhiwei Zhang, Changhua Wang, Yan Su, Chenjuan Ye, Rui Pan, Xiang Liu, Xianyou Qu, Xiaozhong Lan

**Affiliations:** a Chongqing Key Laboratory of Traditional Chinese Medicine Resource, Chongqing Academy of Chinese Materia Medica, Chongqing, China; b TAAHC-SWU Medicinal Plant Joint R&D Centre, Tibetan Collaborative Innovation Centre of Agricultural and Animal Husbandry Resources, Food Science College, Tibet Agriculture & Animal Husbandry University, Nyingchi, China

**Keywords:** *Saussurea medusa*, chloroplast genome, phylogenetic analysis

## Abstract

*Saussurea medusa* is an important traditional Tibetan medicinal plant in China. In this study, we assembled the complete chloroplast (cp) genome of *S. medusa*. The complete *S. medusa* chloroplast genome is a circular molecular structure of 152,257 bp in length with coding GC 37.93%, consisting of two inverted repeats (25,204 bp) separated by a large single-copy region (83,334 bp) and a small single-copy region (18,515 bp). The complete chloroplast genome of *S. medusa* contained 130 genes, including 87 protein-coding genes, 35 tRNA genes, and eight rRNA genes. Phylogenetic analysis shows that *S. medusa* is most closely related to *Saussurea inversa* and *Saussurea pseudoleucoma*. The complete chloroplast genome sequence of *S. medusa* facilitates the phylogenetic studies of Asteraceae.

*Saussurea medusa* Maxim. (Maximowicz 1881) belongs to the *Saussurea* Candolle, Asteraceae family. There are 289 species (191 endemics) of *Saussurea* in China (Wu et al. 2011). The plant has been commonly used as a traditional Tibetan medicine for the treatment of inducing abortion, nourishing blood, detoxifying (Wei et al. 2020). At present, the research of *S. medusa* mainly focuses on chemical composition, pharmacological action, and cell culture. However, there are few studies on chloroplast genes. So we assembled and characterized the complete chloroplast genome sequence of *S. medusa* to provide information for the identification of *Saussurea* Candolle, as well as assist the further phylogenetic study of Asteraceae.

The total genomic DNA of *S. medusa* was extracted from the fresh leaves that were collected in Jiangda County of Xizang (Tibet) Autonomous Region, China (N32°9′, E97°57′). The voucher samples (542122190722379LY) were deposited at the Herbarium of the Chongqing Academy of Chinese Materia Medica (SM, http://www.cqacmm.com/, Xiang Liu, zysliux@163.com), Chongqing, China. Genomic DNA was extracted by using the modified CTAB method (Doyle and Doyle 1987). Total DNA was used for the shotgun library construction. After cluster generation, libraries were sequenced on an Illumina Novaseq 6000 platform and 150 bp paired-end reads were generated. About 3.0 G base pairs of sequencing data in total were obtained and then *de novo* assembled using the program GetOrganelle v1.5 (Jin et al. 2018). The chloroplast genome annotation was performed through the online program GeSeq (Tillich et al. 2017) and CPGAVAS2 (Shi et al. 2019), followed by manual correction. The annotated genomic sequence has been registered into GenBank with the accession number (MZ128902).

The complete *S. medusa* chloroplast genome is a circular molecular structure of 152,257 bp in length with coding GC 37.93%, consisting of two inverted repeats (25,204 bp) separated by a large single-copy region (83,334 bp) and a small single-copy region (18,515 bp). The complete chloroplast genome of *S. medusa* contained 130 genes, including 87 protein-coding genes, 35 tRNA genes, and eight rRNA genes.

To investigate the phylogenetic relationship of *S. medusa*, the phylogenetic tree was generated by the maximum likelihood (ML) method from alignments created using the MAFFT v7 (Katoh et al. 2017), and RAxML (v8.2.10) (Stamatakis 2014). We selected twenty published complete chloroplast genomes from GenBank to assess the genetic and phylogenetic relationship with *S. medusa* (19 Asteraceae and one Campanulaceae). As shown in the phylogenetic tree (Figure 1), *S. medusa* is most closely related to *S. inversa* and *S. pseudoleucoma*. The result was consistent with the traditional plant morphological taxonomy. The complete chloroplast genome sequence of *S. medusa* facilitates the phylogenetic studies of Asteraceae.

**Figure 1.**
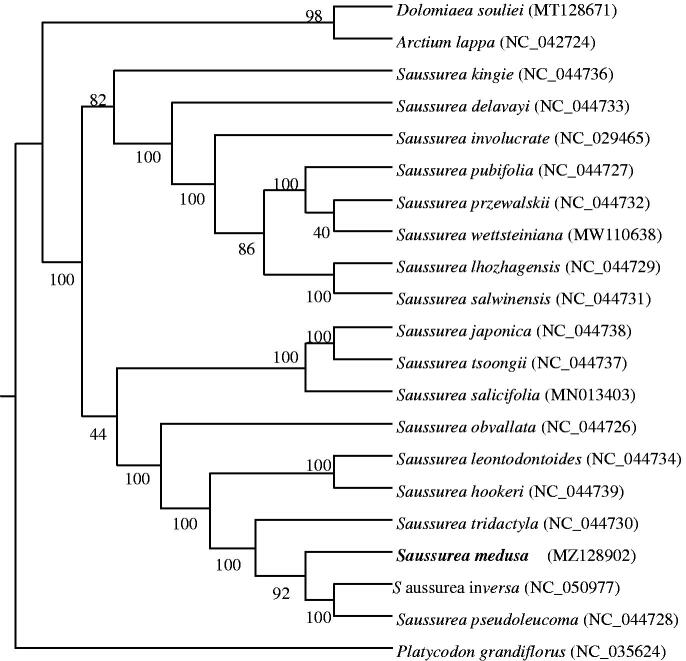
The maximum-likelihood tree is based on *Saussurea medusa* and related chloroplast genomes. The genomes accession number is a list in the figure. Bold shows our newly assembled chloroplast genome of *Saussurea medusa*. The bootstrap value based on 1000 replicates is shown on each node.

## Data Availability

The genome sequence data that support the findings of this study are openly available in GenBank of NCBI (https://www.ncbi.nlm.nih.gov/) under the accession MZ128902. The associated BioProject, SRA, and Bio-Sample numbers are PRJNA728186, SRR14470736, and SAMN19071285, respectively.
